# It is Time to Screen for Homozygous Familial Hypercholesterolemia in the United States

**DOI:** 10.5334/gh.1316

**Published:** 2024-05-03

**Authors:** Samuel S. Gidding, Christie M. Ballantyne, Marina Cuchel, Sarah de Ferranti, Lisa Hudgins, Allison Jamison, Mary P. McGowan, Amy L. Peterson, Robert D. Steiner, Melissa K. Uveges, Yunshu Wang

**Affiliations:** 1 World Heart Federation, US; 2 Geisinger Health, US; 3 Baylor College of Medicine, US; 4 Perelman School of Medicine, University of Pennsylvania, US; 5 Boston Children’s Hospital, US; 6 Rogosin Institute, US; 7 Weill Cornell Medicine, US; 8 Family Heart Foundation, US; 9 Dartmouth Hitchcock Medical Center, US; 10 University of Wisconsin School of Medicine and Public Health, US; 11 Leadiant, Mirum, PTC-Consultant, PreventionGenetics, part of Exact Sciences-Employee with equity, University of Wisconsin School of Medicine and Public Health, US; 12 Boston College, Connell School of Nursing, US; 13 World Heart Federation, CH

**Keywords:** Homozygous Familial Hypercholesterolemia, Best Screening Practices, Universal, Newborn Screening

## Abstract

Homozygous familial hypercholesterolemia (HoFH) is an ultra-rare inherited condition that affects approximately one in 300,000 people. The disorder is characterized by extremely high, life-threatening levels of low-density lipoprotein (LDL) cholesterol from birth, leading to significant premature cardiovascular morbidity and mortality, if left untreated.

Homozygous familial hypercholesterolemia is severely underdiagnosed and undertreated in the United States (US), despite guidelines recommendations for universal pediatric lipid screening in children aged 9–11. Early diagnosis and adequate treatment are critical in averting premature cardiovascular disease in individuals affected by HoFH. Yet, an unacceptably high number of people living with HoFH remain undiagnosed, misdiagnosed, and/or receive a late diagnosis, often after a major cardiovascular event.

The emergence of novel lipid-lowering therapies, along with the realization that diagnosis is too often delayed, have highlighted an urgency to implement policies that ensure timely detection of HoFH in the US. Evidence from around the world suggests that a combination of universal pediatric screening and cascade screening strategies constitutes an effective approach to identifying heterozygous familial hypercholesterolemia (HeFH). Nevertheless, HoFH and its complications manifest much earlier in life compared to HeFH. To date, little focus has been placed on the detection of HoFH in very young children and/or infants.

The 2023 Updated European Atherosclerosis Society Consensus Statement on HoFH has recommended, for the first time, broadening pediatric guidelines to include lipid screening of newborn infants. Some unique aspects of HoFH need to be considered before implementing newborn screening. As such, insights from pilot studies conducted in Europe may provide some preliminary guidance.

Our paper proposes a set of actionable measures that states can implement to reduce the burden of HoFH. It also outlines key research and policy gaps that need to be addressed in order to pave the way for universal newborn screening of HoFH in the US.

## Introduction

Homozygous familial hypercholesterolemia (HoFH) is an inherited condition caused by the presence of biallelic pathogenic variants in genes impacting low density lipoprotein (LDL) receptor function and causing extremely elevated LDL-cholesterol [1]. Historically, HoFH was defined phenotypically as a combination of extremely elevated LDL-cholesterol, typically >400–500 mg/dL, in association with a family history of elevated cholesterol or premature coronary heart disease in both parents. In the genomic era, the term HoFH encompasses individuals with extremely elevated LDL-cholesterol related to LDL receptor dysfunction caused by biallelic pathogenic variants in *LDLR, APOB, PCSK9*, or *LDLRAP1*. *LDLR* variants are a much more common cause than variants in *APOB, PCSK9*, or *LDLRAP*.

### Public health importance

Homozygous familial hypercholesterolemia causes severe cardiovascular morbidity characterized by atherosclerotic heart disease and/or aortic stenosis that often presents in childhood. Morbidity and mortality have been reported in the first decade of life. Homozygous familial hypercholesterolemia impacts about one in 300,000 people; a diagnosis can be confirmed relatively easily, utilizing physical examination and existing tests: lipid profiles and genetic testing. However, HoFH diagnosis is typically delayed, not occurring until late childhood or adolescence in the most severe cases, and young to mid adulthood in those with lower, but still extreme elevations of LDL-cholesterol. Lack of physical findings or their recognition, further contributes to delayed diagnosis [1,2,3]. Homozygous familial hypercholesterolemia is different from heterozygous familial hypercholesterolemia (HeFH) in that morbidity and mortality occur much earlier in life and some of the manifestations, such as aortic stenosis, once developed, tend to progress, even in the setting of optimal therapy.

### Treatment

Current guidelines recommend treatment begin at the time of diagnosis, as opposed to 8–10 years of age, as in HeFH. This treatment typically requires care by expert professionals and a personalized medicine approach, informed by genetic diagnosis, response to medication, resources available in different care settings, and patients’ individual circumstances [1,4,5]. Decades of increased survival have been documented in published cohorts of HoFH patients managed according to historic treatment regimens including lipoprotein apheresis, statins, ezetimibe, other medications, and liver transplantation [3,6]. Substantial morbidity and mortality are preventable with prompt diagnosis. Because of this, and the large number of resources required to care for HoFH, the Global Call to Action on FH recommends that HoFH receive special consideration [7].

With the recent introduction and approval of new and novel lipid lowering therapies, most importantly those that do not require functioning LDL receptors for efficacy, new hope exists for those living with HoFH [1,4]. However, these new treatments have not yet been tested in those under five years of age, a group which might benefit the most but in whom data on safety and efficacy are urgently needed.

### Time to act

The existence of these new agents, along with the realization that diagnosis is often severely delayed, has stimulated the need to address the public policy gap related to delayed identification of HoFH in the United States (US). As yet, we are not aware of a single HoFH patient in the US who has followed the ideal path of newborn identification, followed by lifetime treatment to prevent the cardiovascular complications of FH. This paper will address the lived experience perspectives on HoFH, lipid screening strategies that would facilitate HoFH recognition, the possibility of newborn screening, and ethical issues related to HoFH screening. We will conclude with recommendations for policies that can be implemented now and help narrow existing evidence gaps.

## The Life Course of HoFH: The Lived Experience

My family has a long and frightening history of early heart disease. Generations of relatives on my father’s side died far too young of heart attacks. Still, we were relatively lucky. Early on, doctors recognized that the cause of these early deaths in the family had to be genetic. Eventually, the deaths were even tied to ‘genetic high cholesterol.’ When my father had his first heart attack at 28, no one was even surprised. My mother’s family, on the other hand, had a less remarkable health history. Most deaths seemed common and expected. My maternal grandfather had a heart attack in his early fifties, but non-genetic factors like smoking and stress were cited as causes. My parents understood, from family experience, it was possible my dad could pass this condition on to their children, but no one discussed with them what that risk could look like.

When I developed ‘bumps’ on my knees and elbows at the age of three, my parents were not overly concerned. The first doctor I saw was a dermatologist, who suggested molluscum contagiosum. A few years later, when I was five, my parents decided to check cholesterol levels for all three of their children. My total cholesterol was close to 900 mg/dl. My brother and sister both had high cholesterol as well, but not like mine. All three of us were diagnosed with FH but given the apparent lack of evidence for FH on my mother’s side, we were told I had ‘severe FH’. And that was the diagnosis we lived with. Several years later, at age 13, I was diagnosed with supravalvular aortic stenosis. No one suggested this could be related to my FH.

I was put on bile acid sequestrants and statins starting at age ten, but no treatment got my LDL under 400 mg/dl. At 28, I underwent my first bypass surgery and aortic valve replacement. A year later, under the care of a high-risk obstetrician and with the knowledge, if not support, of my cardiologist, I became pregnant with my first child. I was told I had a 50% chance of passing FH on to my child. I was treated with LDL apheresis during pregnancy, and eventually delivered via emergency C-section at 31 weeks as my body struggled to support the pregnancy. My son’s cholesterol was tested, at my insistence, at his two-year-old check-up, but never brought up by his pediatrician.

The complications from my first pregnancy convinced us that adoption was a better choice to grow our family, and we brought my daughter home a few years later. Shortly after she came home, when I was 35, I suffered a heart attack and sudden cardiac arrest during a stress echo and underwent a second bypass. At that point, I was feeling lost and desperate. I asked my cardiologist to please try to find me a new treatment – I felt there had to be more we could do to get my cholesterol down. That doctor found a clinical trial that was starting nearby, and as part of the screening for participation in the trial, I had a genetic test that confirmed my actual diagnosis – HoFH. Despite the fact that I had known about FH my whole life, my world was rocked.

How had it taken this long to get the right diagnosis? Looking back, I seemed to display every indication of HoFH, it was just the health history of my mother’s family that did not seem to fit expectations. I had a caring team of physicians, and yet still the diagnosis was missed. I do feel fortunate that despite the missing diagnosis, I still was treated for FH. But there were so many years lost when I could have been treated more aggressively, been part of more trials, or just simply been aware of what my future could hold. I could have known that my son would definitely inherit FH, which would have helped me push his treatment as well.

But finally, with the correct diagnosis, I understood why I wasn’t having the same success on statins my siblings had had, and I finally felt empowered to push for more and better treatment with my medical team. I have no doubt that for HoFH, early diagnosis matters.

## Current Status of HoFH Care

Since 2022, data from three HoFH registries has been published [2,3,8]. The largest of these is the HoFH International Clinical Collaborators Registry that included 751 patients from 38 countries and 88 institutions using a retrospective cohort study design [3]. Two other registries, CASCADE (Cascade Screening for Awareness and Detection) FH^Ò^ Registry from the US and the national FH Canada registry,^1^ reported on the longitudinal experiences of 67 and 48 HoFH patients, respectively [2,8]. All three registries document the burden of premature Atherosclerotic cardiovascular disease (ASCVD) that persists in the HoFH population and highlight the consequences of late diagnosis.

One of the most important findings from the HoFH International Clinical Collaborators Registry was the presence of early cardiovascular disease; coronary artery interventions were reported to occur as early as age four, angina pectoris, coronary artery bypass grafting, and aortic valve replacement at age five, and myocardial infarction at age 10 [3]. Individuals from non-high-income countries were less likely to receive three or more lipid lowering agents, were less likely to achieve guideline recommended LDL-C levels and, not surprisingly, the age of first major adverse cardiovascular event came more than a decade earlier. Individuals from non-high-income countries had significantly higher median untreated LDL-C levels and were considerably younger at diagnosis than from low-income countries [3].

The CASCADE FH Registry reported on 67 adults (n = 51) and children (n = 16) [2]. One of the most striking results from this registry, and confirmed by the other registries, was the significantly higher untreated LDL-cholesterol levels found in patients enrolled in the registry as children compared with those enrolled as adults (median [IQR] 776 mg/dL [704–892] in children versus 533 mg/dL [467–702] in adults), a difference which persisted despite treatment. This suggests that only the most severely impacted patients are diagnosed in childhood. At enrollment 78% of adults and 44% of children already had coronary artery disease with the median age of onset in adults being 30.5 (21.1–41.0) and 8.9 (4.5–10.7) in children. Compared to either the CASCADE FH Registry cohort or the HoFH International Clinical Collaborators Registry, those from the Canadian registry had a greater burden of both aortic stenosis and aortic valve replacement [2,3,8].

All registries document the difficulty of achieving treatment goals for LDL-cholesterol defined by existing evidence-based guidelines (<100 mg/dL in the absence of coronary heart disease and <70 mg/dL with existing disease) [2,3,8]. Recommended treatments, beginning with rapid up-titration of statins and ezetimibe, followed by introduction of specialized treatments such as Proprotein convertase subtilisin/kexin type 9 (PCSK9) inhibitors, Angiopoietin-like 3 (ANGPLT3) inhibitors, and lomitapide may help patients get to goal. If not, lipoprotein apheresis, if available, is indicated. In children, liver transplantation may be a reasonable alternative for severe cases [4,5]. The hope for successful gene therapy or gene editing treatments with CRISPR or other technology exists [9].

## Current FH Screening Practices

Current recommendations for FH screening are built upon the original recommendations of Wilson and Jungner as recently adapted in a systematic review and consensus document [10,11,12]. These are summarized in Table 1. In Europe, only Slovenia and Slovakia have nationwide universal FH screening programs at ages 5 and 11 [12]. This is despite support by the European Commission Public Health Best Practice Portal for pediatric FH screening. Current guidelines from several US medical and advocacy organizations recommend universal pediatric lipid screening at ages 9–11 years [13,14]. Recent Chinese guidelines also recommend universal screening of children during school entry physical exams [15]. Screening practices that separate HoFH from HeFH have not been addressed in any guideline.

**Table 1:** HoFH satisfies conditions for screening.


SCREENING CRITERIA	EVIDENCE

HoFH is an important public health problem	Frequency of 1:300,000 Early treatment extends life expectancy substantially

HoFH natural history is well understood	Case series cumulatively including over 1000 patients exist

There is a suitable, acceptable diagnostic test	Lipid levels Genetic testing

Treatment recommendations and acceptable treatments exist	Many existing guidelines worldwide Clinical trials and case series of most available treatments and new treatments in trials

Facilities for diagnosis and treatment exist	Lipid specialty clinics exist across the United States supported by professional organizations and advocacy groups

Cost is acceptable to society	HoFH care is currently reimbursed by existing public and private insurance policies


Cascade screening of first-degree and second-degree relatives of FH probands is the most common form of screening worldwide, with well-developed programs in several countries (the Netherlands, Norway, Spain, Brazil) and others developing programs. In addition to many countries in Europe, pediatric and adult lipid clinics with FH case detection and cascade screening/recommendations exist in Australia, Canada, Japan, Columbia, Brazil, Uruguay, Iraq, Oman, and China but there is little focus on the detection of HoFH in young children [7,16]. Japanese guidelines recommend lipid screening before age 10 if both parents have HeFH [17]. The Wits Find-FH Program in South Africa demonstrates the value of FH case finding and cascade lipid screening in diverse ancestries with a high prevalence of HoFH due to strong founder effects, but data are sparse, particularly for Black South Africans [18]. Although many organizations recommend genetic screening in suspected FH individuals and their first-degree relatives as the best approach for early, accurate diagnosis of FH, none recommended testing timed to very early childhood, i.e., prior to age two, and targeted to offspring with HeFH parents [19].

The 2023 updated European Atherosclerosis Society Consensus Statement on HoFH was the first document to make formal screening recommendations specific to HoFH [1]. The first recommendation was to screen individuals of any age who meet clinical criteria for HoFH, e.g., skin xanthomas, angina, aortic valve disease, or very high LDL-C/premature CVD in both parents. The second was to expand pediatric guidelines to include screening of newborn infants when both parents have very high LDL-cholesterol levels or are from regions with a strong founder gene effect. The third was to establish more national pediatric universal cholesterol screening programs.

Novel cascade screening programs have involved child – parent screening at the time of routine immunizations in one-year-olds [20]. A benefit of this program is the identification of unrecognized young parents and other blood relatives with FH. One small pilot study in the US utilized the Family Heart Foundation as the coordinating center to reach out to family members of genetically confirmed HeFH probands [21]. In this program, at baseline 18% of families had already been screened; after engagement with the Family Heart Foundation 55% of families were willing to be screened. This program is now being tested on a larger scale through a federal grant.

## Preconception, Prenatal, and Pre-Implantation FH Screening

Guidelines are lacking for the preconception screening of couples with FH who are considering pregnancy. An expectant parent with HeFH has a 25% chance of their child inheriting HoFH if the partner also has HeFH and even higher risks if the second parent or both parents live with HoFH. This knowledge may influence the decision to have a child or to seek prenatal or pre-implantation genetic testing. If not performed preconception, lipid screening in pregnant persons might be possible in the first few months of pregnancy before the normal increase in lipids that occurs later during the gestation, potentially confounding screening results [22]. Knowledge of the diagnosis of FH prior to pregnancy is also important because of the potential for undiagnosed significant coronary artery disease and increased risk associated with pregnancy. To assess the chance of HoFH in offspring, screening of the partner should be performed, especially when there is a family history of hyperlipidemia or premature coronary artery disease, consanguinity, or from a region with an FH founder effect. The diagnosis of FH should be confirmed by genetic testing with appropriate pre- and post-test counseling [19,23]. FH screening could also be considered as part of an expanded genetic carrier screen that is currently offered in selected settings, though equity concerns for this approach exist [24].

Knowledge of a high probability of HoFH in the newborn infant would prompt lipid and/or genetic testing of cord blood at birth followed by referral to a pediatric lipid specialist for early diagnosis and treatment. Alternatively, prenatal screening and subsequent procedures that could influence reproductive decisions and management may be available [25], including chorionic villus biopsy, amniocentesis, and preimplantation genetic testing prior to assisted reproductive technology implanting unaffected embryos. A recent publication described the very personal and difficult decisions made by two women with HeFH about the initiation and continuation of their pregnancies and offered several recommendations for clinicians [26].

## Newborn Screening

Newborn screening has been widely touted as one of the most successful public health measures of the past half century [27]. The goal of newborn screening is to identify serious and potentially treatable conditions that might not be easily identifiable by signs and symptoms early in life in order to reduce or prevent morbidity and mortality. Selection of disorders to be included in newborn screening programs varies by state in the US, and outside of the US, by country. Currently in the US, each state decides which disorders are included in newborn screening in that state; generally, this is written into state law.^2^ The Recommended Uniform Screening Panel (RUSP) includes disorders that the United States Secretary of the Department of Health and Human Services recommends for screening. The selection of conditions has been informed by a document commissioned by the Health Resources and Services Administration [28]. More specifically, disorders on the RUSP are chosen based on the evidence that supports a potential benefit of screening, ability of states to screen for the disorder, as well as availability of treatments. The Advisory Committee on Heritable Disorders in Newborns and Children reviews nominations of conditions to be considered for the RUSP, arranges evidence reviews, and after further consideration makes recommendations to the Secretary. States are not bound by the RUSP but many states attempt to match the RUSP.

As with all disorders, there are unique aspects of HoFH to be considered in the implementation of universal newborn screening. Of the Wilson and Jungner criteria, the most challenging principles to meet for HoFH newborn screening include the existence of a suitable, acceptable test; facilities for diagnosis and treatment; and cost-balancing. In addition, while treatments exist, because of delayed identification and exclusion of very young children from clinical trials, there is limited treatment experience in children under five years of age with no reports of children identified at birth.

Currently there is no validated newborn screening test for HoFH. As individuals affected by HoFH would exhibit the highest levels of candidate biomarkers, such as cholesterol or apolipoprotein B (apoB), tests designed to screen for HeFH would likely fulfill this purpose. Those infants with biomarker levels above a threshold on screening tests would require genetic testing to confirm the diagnosis. Individuals with HoFH and severe HeFH would likely be identified.

Successful efforts to modify commercially available assays for quantification of cholesterol, apolipoprotein B, and lipoprotein-cholesterols for high-throughput analysis of dried blood spots towards development of newborn screening for HeFH have been published [29]. Assay validation results were within acceptable limits for newborn screening. LDL-C and apoB distribution curves by the same assays support candidacy for newborn screening [30]. A pilot study screening newborns for HeFH in the Czech Republic^3^ testing LDL-cholesterol levels on cord blood with reflex genetic testing is underway, a screening program in Germany is measuring total cholesterol levels in newborns and lipid distributions from birth to early in the second year of life in Denmark have been published [31]. These are important first steps towards validation and implementation of a newborn screening test for the identification of newborns with HoFH.

## Additional Issues

### Ethics

Screening for HoFH raises issues related to informed consent, privacy and confidentiality, beneficence, and resource allocation. In general, newborn screening is required by state law, therefore informed consent is not typically obtained. However, for genetic testing in children outside of newborn screening, informed consent is often obtained and addresses protections for privacy and confidentiality. Table 2 includes risk-benefit considerations of HoFH newborn screening.

**Table 2:** Risk-Benefit considerations of incorporating HoFH into newborn screening [33].


POTENTIAL BENEFITS	POTENTIAL RISKS

Would increase identification of a condition that causes premature CVD, facilitating early treatment	Evaluation and care plan for phenotype positive/gene negative patients not established

HoFH treatment is highly beneficial	There is little data on HoFH treatment prior to 5 years of age; side effects of most treatments in infancy are unknown.

Identifying HoFH in an infant allows relatives to be screened, improving the health of family members	Screening children to benefit family members shifts emphasis away from health interests of the child alone [34].

Adding HoFH to NBS could simultaneously incorporate screening for severe HeFH	Patients with milder forms of HeFH would not be identified unless genetic screening performed.

Adding HoFH to NBS does not require an additional blood sample, since NBS is already done at birth	

Assays for testing HoFH on the NBS are already available and reagents are low cost	Proposed screening methods not fully validated; full downstream costs of screening and diagnosis are not known.


### Cost

The cost of newborn screening for HoFH has not yet been adequately addressed. Identification of the costs of screening, diagnosis, and treatment would likely require a comprehensive economic analysis. Such analyses have been performed for other disorders and the approach is easily adaptable; Prosser et al. have reviewed important concepts in decision making about newborn screening including cost evaluation [32]. In terms of testing costs, the assays for HeFH referenced above were performed using instrumentation already available within the newborn screening laboratory, and the cost of reagents was minimal (less than tree US dollars per test). The inclusion of ultra-rare disorders such as HoFH has been questioned in decision making for newborn screening. However, many ultra-rare disorders are currently included in the RUSP, some with population frequencies likely less than that of HoFH (see Table 3).

**Table 3:** Select disorders screenable in the newborn period and their estimated prevalence.


DISORDER	ESTIMATED PREVALENCE IN US POPULATION

Heterozygous familial hypercholesterolemia	1 in 300

Phenylketonuria*	1 in 16,200

Maple Syrup Urine Disease*	1 in 150,000

Homozygous familial hypercholesterolemia (high prevalence estimate)	1 in 160,000

Homozygous familial hypercholesterolemia (low prevalence estimate)	1 in 400,000

Glutaric Acidemia Type 2*	1 in 465,000

Carnitine Palmitoyltransferase Deficiency Type 1A*	1 in 500,000


*Disorder currently listed on the Recommended Uniform Screening Panel. This is the list of disorders that the Secretary of the Department of Health and Human Services recommends states screen for as a part of their standard universal newborn screening programs. (https://www.hrsa.gov/advisory-committees/heritable-disorders/rusp).

### Direct-to-consumer testing

Newer commercial services may impact the landscape of lipid testing and diagnosis. These include the opportunity for consumers to obtain their own lipid results via a blood spot, as currently offered by several companies without a physician order, and genetic results returned to consumers as part of a genetic profile purchased by consumers. Although this testing provides the opportunity for cascade testing outside of the medical setting, it is concerning when testing is not performed by certified laboratories, or the return of results does not include genetic counseling. Further, how primary care providers may respond to individuals presenting with direct-to-consumer laboratory results has not been thoroughly studied.

### Centralized resources

Given that patients with HoFH should be under the care of a pediatric lipid specialist, referral networks to expedite HoFH care should exist. Most pediatric lipid specialists are self-identified and come from a variety of disciplines, including cardiology, endocrinology, genetics/metabolic disease, gastroenterology, and those who confine their care to lipid disorders. For geographic reasons, many patients with HoFH may require a hybrid approach to care, with the patient receiving testing and care at their home institution, with consultation and virtual remote care from a more geographically distant specialty clinic. This is specifically true for patients requiring investigational medications, lipid apheresis, or liver transplantation. Planning so that access to these services exists across the US is needed.

### Role of advocacy organizations

Advocacy organizations are important in improving access to care, lobbying for important health policies to guarantee HoFH care, overcoming health disparities, and in connecting individuals affected by HoFH with local or regional specialists and to HoFH-specific clinical trials. Additionally, such organizations provide emotional support and forums for people living with and families impacted by HoFH to meet and share experiences. In the US, the Family Heart Foundation currently provides all of these services but is dependent on fundraising to sustain these programs and services.

## Recommendations

In this document, we provide the rationale for immediate action necessary to improve HoFH identification and treatment. Too many patients suffer premature morbidity and mortality due to delayed recognition. This is now particularly concerning given the plethora of new therapies available and the opportunities for personalized approaches to care based on understanding of genetics.

**Figure 1:**
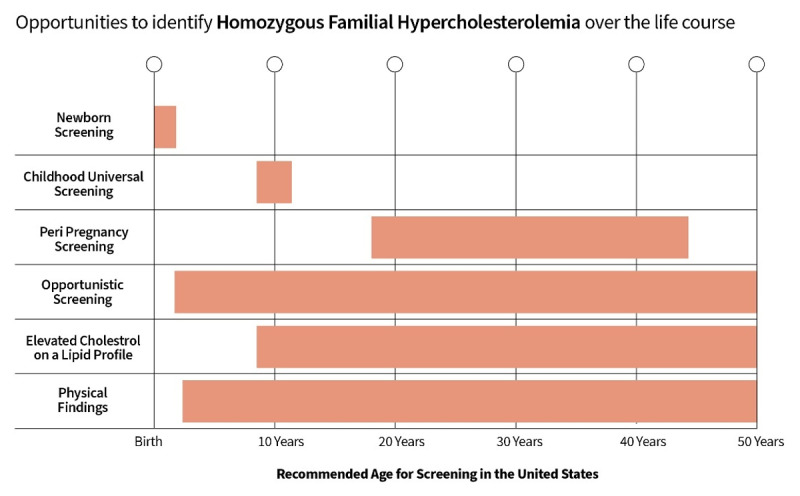
Opportunities to identify HoFH over the life course.

Actions to improve HoFH identification can be divided into two categories: strategies which could be implemented immediately and those which require further research and evidence evaluation. These actions are summarized in the Central Illustration (Figure 1).

The first step would be to be follow existing US multi-society guidelines for lipid screening, as opposed to US Preventive Services Task Force, making sure everyone eligible for a lipid profile has received one. This includes all people between the ages of 9–11 years and all those over the age of two years with a family history suggestive of FH or premature ASCVD. These guidelines can be extended to infancy for those with a family history consistent with HoFH (both sides of the family with suggestive histories) and prenatal screening of the second parent if one parent has FH and prenatal genetic testing in high-risk situations. Laboratory reports should be standardized so that all patients with an LDL-cholesterol above 400 mg/dL include an alert regarding the need for an evaluation for HoFH in the absence of secondary cause for extreme hypercholesterolemia. Independent of these actions, many steps can be taken to improve HoFH care in general and facilitate recognition and treatment (Table 4).

**Table 4:** Facilitating HoFH care.


MEASURES	RATIONALE

Provide education to family medicine physicians, pediatricians, internists, obstetricians, nurse practitioners and physician assistants regarding the value of screening for HoFH in proper settings and recognition of physical findings	Both HeFH and HoFH are under-diagnosed and under-treated.

Guarantee health care coverage for all HoFH patients	HoFH is a rare disease with high lifetime cost for life-saving health care.

Create a separate diagnostic code for HoFH	Allows tracking of those with the condition.

Establish referral networks nationwide for HoFH care that include shared care between home facilities and specialized care sites	Will improve delivery of evidence-based health care nationally; facilitates the development of a HoFH registry and research.

Educate the public about the value of 1) Lipid testing to identify actionable LDL-C levels and to improve cardiovascular health and 2) Genetic testing for Tier 1 conditions, including FH, to improve acceptability; remove discriminatory barriers in society to such testing	Overcome barriers to the public’s understanding of FH as a condition and the understanding that lipid control is important for long term health.


To truly identify all individuals with HoFH at an early age and to maximize the benefit of early treatment, newborn screening for the condition is the optimal course of action. A population-based screening program would overcome disparities related to access to care, health insurance, and health literacy. To achieve this, many evidence and policy gaps must be closed. These include establishing the best method for newborn screening, advocating for newborn screening for HoFH in the US at the national and state levels, establishing pilot programs to understand best practices, and obtaining clinical data on treatment of children prior to five years of age to establish best practices. Regulatory agencies should understand the value of including the youngest patients with the condition in clinical trials, after safety of investigational agents in older children has been established, to further encourage early identification.

## Footnotes

www.FHCanada.net.

https://www.babysfirsttest.org/.

https://clinicaltrials.gov/study/NCT05638022.
